# Early Postoperative Changes in Macular Choroidal Thickness After Uncomplicated Phacoemulsification in Patients With and Without Glaucoma: A Swept-Source Optical Coherence Tomography Study

**DOI:** 10.7759/cureus.45822

**Published:** 2023-09-23

**Authors:** Konstadinos Sotiropulos, Dimitrios Kourkoutas, Klio I Chatzistefanou, Konstantinos Droutsas, Marilita M Moschos

**Affiliations:** 1 Department of Ophthalmology, 417 Army Shared Fund Hospital, Athens, GRC; 2 Department of Ophthalmology, 401 General Military Hospital of Athens, Athens, GRC; 3 First Department of Ophthalmology, National and Kapodistrian University of Athens, Athens, GRC

**Keywords:** choroidal thickness changes, swept-source optical coherence tomography, glaucoma, phacoemulsification cataract surgery, choroidal thickness, subfoveal choroidal thickness

## Abstract

Purpose

The objective of this study was to examine the impact of uncomplicated phacoemulsification on macular choroidal thickness (CT) within the first three postoperative months and to investigate its relationship with postoperative cystoid macular edema (CME) in both glaucomatous and healthy subjects, utilizing swept-source optical coherence tomography (SS-OCT).

Methods

The non-randomized prospective study involved 82 patients, selected via convenience sampling from the First Department of Ophthalmology, Medical School of Athens, “G. Gennimatas” Hospital, Athens, Greece, between May 2018 and May 2022, undergoing phacoemulsification and intraocular lens (IOL) implantation. The inclusion criteria encompassed patients aged 50 years or above, with or without glaucoma. Patients with ocular pathologies that could influence macula or CT measurements were excluded. Data collection focused on retinal and CT variables of the macular area, measured using SS-OCT. Baseline measurements were established preoperatively, with follow-up assessments at one week, one month, and three months postoperatively to monitor CT and macular edema onset.

Results

A total of 82 eyes from 82 patients with a mean age of 79.1±8.3 years were included. The study population was divided into a glaucoma group (n=28 eyes) and a control group (n=54 eyes). Our findings indicate a consistently significant increase in macular CT measurements one month after cataract surgery, observed in both glaucomatous and non-glaucomatous eyes. In the first postoperative week, statistically significant changes in CT were observed only in patients with CME. Subsequently, at one-month interval, both patient groups, those with and without CME, exhibited statistically significant changes in CT across all macular sectors. CME was detected in 10 out of 28 eyes in the glaucoma group and in 16 out of 54 eyes in the control group. When evaluating the impact of postoperative CME on groups of glaucomatous and non-glaucomatous eyes, it was observed that glaucomatous eyes exhibited a significantly larger magnitude of change in subfoveal CT (SFCT) (p=0.03) at one month (relative to baseline) compared to non-glaucomatous eyes. There was also a 31% increase in the odds of developing CME for glaucoma patients; this result was not statistically significant (odds ratio {OR}, 1.31; 95% confidence interval {CI}, 0.50-3.47; p=0.57).

Conclusions

During the early postoperative period, the study revealed a significant increase in CT at one month after phacoemulsification in both glaucomatous and non-glaucomatous eyes. When CME was present, a significantly more pronounced magnitude of change in SFCT was observed at one month in glaucomatous eyes, as opposed to non-glaucomatous eyes. This observation suggests a possible selective susceptibility of glaucomatous eyes in the early postoperative period that requires further research.

## Introduction

Phacoemulsification with intraocular lens (IOL) implantation is the most common procedure performed today, resulting in excellent anatomic and functional outcomes and a relatively low complication rate [1]. This surgical procedure releases inflammatory mediators and leads to the breakdown of the blood-aqueous and blood-retina barrier [2,3]. As a result, a local choroidal inflammatory response contributes to cystoid macular edema (CME)[4], as well as choroidal thickening [5]. Several studies have assessed the influence of cataract surgery to subfoveal choroidal thickness (SFCT) with contradictory results [6-8].

Glaucoma is a multifactorial chronic optical neuropathy characterized by functional visual field (VF) defects and changes to the optical nerve structure. Despite the fact that raised intraocular pressure (IOP) remains a major risk factor [9], researchers are also investigating the choroid’s potential role in the development of glaucoma [10]. The choroid is a dynamic tissue of the eye that accounts for approximately 70%-80% of the ocular blood flow [11]. Vascular factors may play a significant role in the pathogenesis of specific types of glaucoma, such as normal-tension and angle-closure glaucoma [12]. Moreover, investigators have displayed reduced choroidal thickness (CT) in normal-tension glaucoma patients when compared to individuals with healthy eyes [13]. Reports indicate that glaucomatous eyes demonstrate a reduction in the average or regional peripapillary CT [14], as well as a decrease in the density of larger choroidal vessels and choriocapillaris [15]. Choroidal thinning has also been connected to a reduction in the innermost choroidal vessels in primary open-angle glaucoma (POAG) [16]. Initial histological examinations provided evidence of a link between the structure of the choroid and glaucoma, as numerous reports indicated a thinner choroid in the eyes with glaucoma compared to those without the condition [14,16]. However, it remains unclear whether choroidal thinning is a cause or a result of glaucoma.

In the last 15 years, there have been significant progressions in optical coherence tomography (OCT), including enhanced depth imaging (EDI) [17], which is a modified version of spectral domain OCT (SD-OCT), and swept-source OCT (SS-OCT) [18], that enable the in vivo noninvasive visualization of the choroid, as well as the quantitative and accurate analysis of both retinal and choroidal layers. Changes in CT measurements have been linked to various ocular conditions, including central serous chorioretinopathy and macular degeneration [18,19]. Additionally, CT alterations have been observed following ocular surgeries [20,21]. Our recent scoping review identified a gap in the existing literature concerning the effects of cataract surgery on macular CT after phacoemulsification in the eyes with glaucoma [22].

Therefore, the aim of the current study was to assess the early alterations in macular CT within three months following phacoemulsification in the eyes with and without glaucoma, utilizing the SS-OCT method, and to ascertain whether these changes in CT are associated with the occurrence of postoperative CME.

## Materials and methods

This prospective, non-randomized study was carried out at the First Department of Ophthalmology, Medical School of Athens, “G. Gennimatas” Hospital, Athens, Greece, between May 2018 and May 2022. The study received approval from the Institutional Review Board of “G. Gennimatas” Hospital, Athens, Greece (approval number: 22272/11-7-2018), and was conducted in compliance with the principles outlined in the Declaration of Helsinki. All participants provided written informed consent before participating in the study.

Patients included in this study were those undergoing uncomplicated cataract surgery (phacoemulsification) and IOL insertion, aged 50 years or older, and with or without a diagnosis of glaucoma. The exclusion criteria included patients with any retinal pathology and systemic disease or systemic medication that could affect retinal or choroidal thickness. The eyes with a history of surgeries or laser treatments were also excluded from the study.

The eyes were categorized into two groups: the control group, comprising healthy eyes, and the glaucoma group, consisting of eyes with glaucoma [23]. This classification was determined based on the results of VF tests and the ONH appearance as established in our prior study [24]. The control group consisted of subjects who satisfied all the following criteria in both eyes: (1) no previous intraocular surgery, (2) IOP of ≤22 mmHg, (3) clinically normal disc appearance, (4) a normal VF result defined as a mean deviation (MD) and pattern standard deviation (PSD) within 95% confidence intervals (CIs) and a glaucoma hemifield test (GHT) result within normal limits (WNL), and (5) no other significant ophthalmic findings. The eyes were categorized as glaucomatous when there were glaucomatous structural damage (neuroretinal rim notching or thinning or the presence of a retinal nerve fiber layer {RNFL} defect) and associated repeatable (≥2 consecutive) VF defects. Glaucomatous VF defect was defined [25] by a GHT outside normal limits (ONL) on at least two VFs or a cluster of three or more non-edge points in a location typical for glaucoma, all of which are depressed on the pattern deviation plot at p<5% level and one of which is depressed at a p<1% level or a PSD with p<5% level.

Each study participant underwent preoperative clinical assessment that encompassed visual acuity testing, Goldmann applanation tonometry, slit-lamp biomicroscopy, and fundoscopy. Furthermore, an experienced ophthalmologist (KS) analyzed the macular area by utilizing the SS-OCT method. Consequently, all patients received uniform treatment, undergoing phacoemulsification cataract surgery performed by the same skilled surgeon (MM). The procedure involved a 2.2 mm clear cornea incision and utilized the Centurion Vision (Alcon Laboratories, Inc., Fort Worth, TX), followed by the implantation of the AcrySof IQ SN60WF (Alcon Laboratories, Inc., Fort Worth, TX) foldable IOL. Postoperative treatment included chloramphenicol and dexamethasone eyedrops. Follow-up examinations included OCT macula scans performed one week, one month, and three months postoperatively.

**Figure 1:**
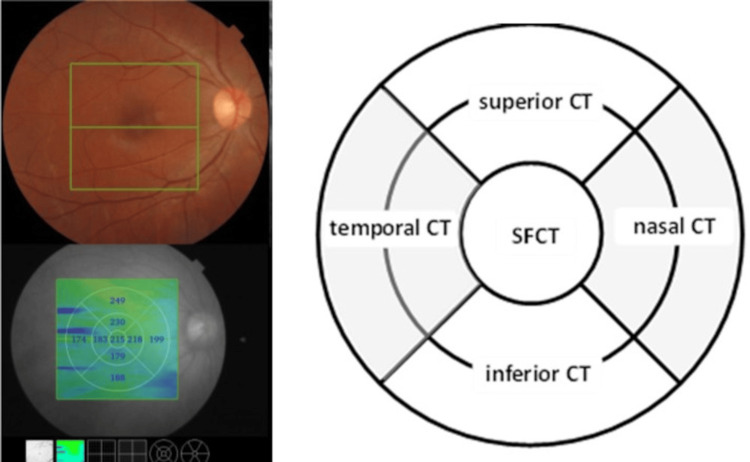
Modified ETDRS grid with five sectors SFCT, subfoveal choroidal thickness; CT, choroidal thickness; ETDRS, Early Treatment Diabetic Retinopathy Study

The Deep Range Imaging (DRI) Triton SS-OCT device (Topcon, Tokyo, Japan) was used to measure the retinal thickness (RT) and CT of the macular area. The DRI Triton SS-OCT utilizes a 1,050 nm wavelength light, with 100,000 A-scans per second, and axial and transverse resolutions of 8 and 20 μm, respectively [26]. Retinal imaging was performed using the three-dimensional (3D) macular cube 7×7 mm scan mode, which featured a 1.3-second scan duration and was centered on the macula. The scan density was set at 512 A-scans×512 B-scans. All DRI OCT images achieved a minimum image quality value (IQV) of 50. The integrated IMAGEnet software (Imagenet LLC, Tampa, FL) automatically conducted the segmentation of each retinal and choroidal junction layer and quantified the thickness of each layer through an automated process. The total retinal thickness of the macula was measured from the internal limiting membrane to the lower border of the retinal pigment epithelium-Bruch’s membrane (BM) complex. The choroidal thickness was measured from Bruch’s membrane (BM), which is the hyperreflective layer on the retina-choroid junction, to the hyperreflective layer on the inner surface of the choroidal-scleral junction (choroidal-scleral interface, CSI) [27]. An average CT measurement for each of the nine macular sectors in the Early Treatment Diabetic Retinopathy Study (ETDRS) grid was provided. The ETDRS nine-pattern grid divides the macula into distinct regions: a central area with a 1 mm radius, an inner ring with a 3 mm radius, and an outer ring with a 6 mm radius. This grid delineates the following nine sectors: the central (subfoveal) sector, inner superior sector, inner nasal sector, inner inferior sector, inner temporal sector, outer superior sector, outer nasal sector, outer inferior sector, and outer temporal sector (see Figure 1). Furthermore, the software automatically computed and displayed the average CT across all nine sectors.

For the purpose of our study, the ETDRS nine-sector grid was modified to include five sectors, as shown in Figure 1. Subsequently, the CT of the central (subfoveal) sector of the macula was defined as the subfoveal choroidal thickness (SFCT). The average CT from the nasal inner and nasal outer sectors was defined as nasal CT. The average CT from the temporal inner and temporal outer sectors was defined as temporal CT. The average CT from the inferior inner and inferior outer sectors was defined as inferior CT. The average CT from the superior inner and superior outer sectors was defined as superior CT.

In our study, we adopted the definition of post-cataract CME as proposed by Kim et al. [28]. Accordingly, postoperative CME was identified as a 40% increase from the baseline thickness of the central sector of the ETDRS grid, as determined by SS-OCT.

Statistical analysis

Continuous variables were expressed as mean±standard deviation, and Student’s t-test was used for group comparison. The chi-square test was used to compare categorical variables. Repeated measure analysis was used to evaluate the impact of phacoemulsification on CT at four different time intervals. The mean differences in the magnitude of CT change between glaucomatous and non-glaucomatous eyes were analyzed using unpaired t-tests. The presence of postoperative CME and glaucoma status were measured as dichotomous variables (the presence or absence of condition). We also calculated the odds ratio (OR) as an estimate of the relative risk for an increase in CT in the eyes with glaucoma by comparing patients with and without CME. All statistical analyses were performed using the Statistical Package for Social Sciences (SPSS) V25 statistical software (IBM SPSS Statistics, Armonk, NY), with p<0.05 set as the threshold for statistical significance.

## Results

Subject demographics

A total of 82 eyes from 82 patients were included, with a mean age of 79.1±8.3 years and a gender distribution of 54.9% female and 45.1% male. The study included 28 patients with glaucoma (study group) and 54 healthy controls (control group). There was no statistically significant difference between the two groups with respect to age, sex, and right/left eye distribution. Preoperative CT measurements in all sectors were found to be significantly larger in glaucoma eyes compared to non-glaucoma eyes. The demographic and clinical characteristics of the participants are presented in Table 1.

**Table 1:** Demographic and ocular characteristics of healthy and glaucomatous subjects included in the study ^1^Data presented as mean±standard deviation ^2^Data presented as number and percentage *P<0.05 was considered significant OD, right eye; OS, left eye; CT, choroidal thickness; SFCT, subfoveal choroidal thickness

	Glaucoma group, n (%)	Control group, n (%)	Overall, n (%)	p-value
Total	28 (100)	54 (100)	82 (100)	
Age (years)^1^	81.2±7.4	78.1±8.6	79.1±8.3	0.109
Eye^2^				0.864
OD	15 (54)	30 (56)	45 (55)	
OS	13 (46)	24 (44)	37 (45)	
Sex^2^				0.217
Male	10 (36)	27 (50)	37 (45)	
Female	18 (64)	27 (50)	45 (55)	
Preoperative CT (µm)^1^				
SFCT	234.75±95.53	183.81±77.20	201.21±86.79	0.019*
Superior CT	236.75±95.18	190.94±74.66	206.59±84.53	0.032*
Inferior CT	217.14±90.91	169.83±67.42	185.99±78.99	0.019*
Nasal CT	214.68±90.87	164.31±68.08	181.51±79.76	0.013*
Temporal CT	239.29±94.31	183.33±72.77	202.44±84.51	0.009*

CT measurements at four different postoperative time intervals

Table 2 shows the mean and standard deviation of CT measurements of both the study (glaucoma) and control (non-glaucoma) groups at four different time intervals (pre- and postoperatively). At the first month, the SFCT and superior and inferior CT were significantly greater than the preoperative values (p<0.05) in both the study and control groups.

**Table 2:** Mean±standard deviation CT (μm) of the study (glaucoma) eyes (n=28) and controls of patients (n=54) at preoperative, week 1, month 1, and month 3 visits P-value (1) compares preoperative with week 1, p-value (2) compares preoperative with month 1, and p-value (3) compares preoperative with month 3 *P<0.05 was considered significant SFCT, subfoveal choroidal thickness; CT, choroidal thickness

Group	Preoperative	Week 1	Month 1	Month 3	p-value (1)	p-value (2)	p-value (3)
SFCT							
Study eye (glaucoma)	234.75±95.53	237.14±100.49	251.89±100.10	237.86±96.49	0.574	0.023*	0.185
Control	183.81±77.20	185.24±79.35	212.24±93.64	190.83±82.11	0.647	0.001*	0.163
Superior CT							
Study eye (glaucoma)	236.75±95.18	233.86±94.57	253.14±99.77	240.21±98.01	0.721	0.025*	0.081
Control	190.94±74.66	192.09±75.99	220.20±104.44	199.81±84.67	0.683	0.004*	0.037*
Inferior CT							
Study eye (glaucoma)	217.14±90.91	217.61±92.61	234.00±90.01	219.89±87.39	0.917	0.027*	0.372
Control	169.83±67.42	171.44±69.41	193.67±86.57	177.04±75.77	0.447	0.001*	0.034*
Nasal CT							
Study eye (glaucoma)	214.68±90.87	216.89±92.75	233.57±101.01	217.75±89.12	0.528	0.064	0.238
Control	164.31±68.08	166.89±68.48	191.74±90.80	173.83±75.24	0.206	0.003*	0.025*
Temporal CT							
Study eye (glaucoma)	239.29±94.31	241.21±100.00	251.64±97.05	241.61±94.06	0.704	0.114	0.347
Control	183.33±72.77	185.09±76.79	208.33±90.85	189.76±79.72	0.579	0.001*	0.080

**Figure 2:**
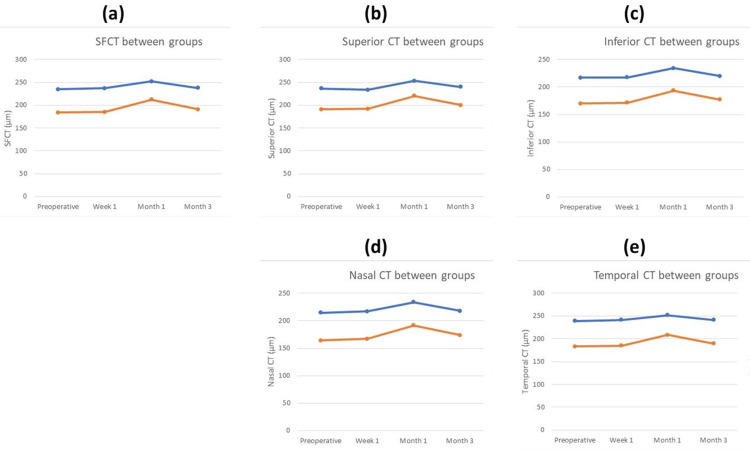
Changes of (a) subfoveal choroidal thickness (SFCT), (b) superior choroidal thickness (CT), (c) inferior CT, (d) nasal CT, and (e) temporal CT at four different time intervals (preoperatively and one week, one month, and three months postoperatively) The data from the glaucoma group is represented using blue lines, while the data from the control group is represented using orange lines

For the same time interval, nasal and temporal CT were significantly greater than the preoperative values only in the control group. Three months postoperatively, there were no significant changes in CT observed in any of the macular sectors in glaucomatous eyes. However, in control eyes, superior, inferior, nasal, and temporal CT remained significantly different. Postoperative changes in CT are graphically presented in Figure 2, which highlights the changes over time in various macular sectors for both the study and control groups.

Postoperative CT changes and the presence of postoperative CME

Table 3 shows the frequency of CME in different study groups. CME was detected in 10 out of 28 eyes in the glaucoma group and in 16 out of 54 eyes in the control group. Our data suggests that glaucoma patients developed postoperative CME 1.31 times more often than healthy subjects. Although there was a 31% increase in the odds of developing CME for glaucoma patients, this result was not statistically significant (odds ratio {OR}, 1.31; 95% CI, 0.50-3.47; p=0.57).

**Table 3:** Frequency of cystoid macular edema (CME) in different study groups (glaucoma and non-glaucoma) The data present is the number of eyes (percent) in each group of eyes

		CME		Total
		Yes	No	
Glaucoma	Yes	10 (12%)	18 (22%)	28 (34%)
	No	16 (20%)	38 (46%)	54 (66%)
Total		26 (32%)	56 (68%)	82 (100%)

Table 4 displays the changes in CT across all macular sectors between the eyes with and without CME. At the first postoperative week, statistically significant changes in CT were observed for all macular sectors except the inferior sector, exclusively in patients with CME.

**Table 4:** Mean±standard deviation CT (μm) of the study (cystoid macular edema {CME}) eyes (n=26) and controls of patients (n=56) at preoperative, week 1, month 1, and month 3 visits P-value (1) compares preoperative with week 1, p-value (2) compares preoperative with month 1, and p-value (3) compares preoperative with month 3 *P<0.05 was considered significant SFCT, subfoveal choroidal thickness; CT, choroidal thickness

Group	Preoperative	Postoperative			p-value (1)	p-value (2)	p-value (3)
		Week 1	Month 1	Month 3			
SFCT							
Study eye (CME)	252.5±109.05	259.86±92.05	293.54±115.12	264.50±107.39	0.048*	0.012*	0.192
Control	177.39±62.18	176.55±64.23	194.32±68.51	180.14±65.34	0.794	0.000*	0.297
Superior CT							
Study eye (CME)	255.92±96.41	261.31±92.05	299.65±122.95	267.65±100.91	0.045*	0.035*	0.158
Control	183.68±67.91	180.84±67.81	199.79±75.31	188.52±74.30	0.541	0.000*	0.007*
Inferior CT							
Study eye (CME)	236.00±96.10	239.19±97.61	269.42±105.43	242.00±99.53	0.322	0.025*	0.402
Control	162.77±57.08	163.07±58.04	178.66±63.51	168.30±60.34	0.907	0.000*	0.000*
Nasal CT							
Study eye (CME)	227.31±98.91	233.00±98.84	270.62±117.21	238.31±97.41	0.045*	0.026*	0.184
Control	160.25±58.92	161.20±58.95	176.04±66.39	165.86±63.19	0.675	0.001*	0.006*
Temporal CT							
Study eye (CME)	254.58±102.45	263.15±105.49	263.12±104.86	263.12±104.86	0.013*	0.016*	0.199
Control	178.23±62.18	176.91±64.72	181.63±65.02	181.62±65.02	0.712	0.003	0.121

At one month, both study groups exhibited statistically significant CT changes across all macular sectors. The eyes without CME displayed significant CT changes in the superior, inferior, and nasal sectors, which persisted three months after surgery.

Table 5 displays the changes in CT of the central sector of the macula (SFCT) among the subgroups of glaucomatous and non-glaucomatous eyes with CME, while Table 6 presents the changes in the same subgroups without CME. The changes in SFCT at the first month were more significant in both non-glaucoma and (p=0.002) and glaucoma (p=0.016) participants with CME. For the subgroup without CME, significant SFCT changes were observed only in healthy participants (p=0.011).

**Table 5:** Mean±standard deviation SFCT (μm) of the study (glaucoma) eyes (n=10) and controls (n=16) of patients with cystoid macular edema (CME) (n=26) at preoperative, week 1, month 1, and month 3 visits P-value (1) compares preoperative with week 1, p-value (2) compares preoperative with month 1, and p-value (3) compares preoperative with month 3 *P<0.05 was considered significant SFCT: subfoveal choroidal thickness

Group	Preoperative	Postoperative			p-value (1)	p-value (2)	p-value (3)
		Week 1	Month 1	Month 3			
Study eye (glaucoma)	235.70±102.55	246.10±110.10	279.90±119.60	257.30±121.80	0.081	0.016*	0.197
Control	201.31±65.42	207.13±65.53	216.56±70.38	207.06±68.18	0.189	0.002*	0.141

**Table 6:** Mean±standard deviation SFCT (μm) of the study (glaucoma) eyes (n=18) and controls (n=38) of patients without cystoid macular edema (CME) (n=56) at preoperative, week 1, month 1, and month 3 visits P-value (1) compares preoperative with week 1, p-value (2) compares preoperative with month 1, and p-value (3) compares preoperative with month 3 *P<0.05 was considered significant SFCT: subfoveal choroidal thickness.

Group	Preoperative	Postoperative			p-value (1)	p-value (2)	p-value (3)
		Week 1	Month 1	Month 3			
Study eye (glaucoma)	218.89±109.87	222.06±114.10	231.33±105.02	228.39±101.87	0.213	0.086	0.354
Control	183.71±76.58	180.82±76.61	212.79±95.32	183.37±76.25	0.537	0.011*	0.917

The magnitude of CT change was calculated for each eye by subtracting the CT value that was measured at different postoperative time intervals from the value measured at baseline (preoperative). The magnitude of CT change from baseline is further detailed in Table 7, for both glaucomatous and non-glaucomatous eyes, both with and without CME.

**Table 7:** Mean±standard deviation of CT change (μm) from baseline, for the central macular sector (SFCT), of glaucoma and non-glaucoma eyes with and without CME at week 1, month 1, and month 3 visits *P<0.05 was considered significant using the unpaired t-test SFCT, subfoveal choroidal thickness; CT, choroidal thickness; CME, cystoid macular edema

	Week 1	p-value	Month 1	p-value	Month 3	p-value
Glaucoma eyes						
With CME (n=10)	10.40±16.71	0.168	44.20±47.30	0.035*	21.60±49.09	0.499
Without CME (n=18)	3.17±10.41		12.44±29.00		9.50±42.29	
Non-glaucoma eyes						
With CME (n=16)	5.81±16.90	0.262	15.25±16.93	0.420	5.75±14.81	0.281
Without CME (n=38)	-2.89±28.65		29.08±66.84		-0.34±20.17	

When CME was present, the magnitude of CT change at the first month (relative to baseline) was significantly larger in glaucomatous eyes compared to non-glaucomatous eyes. However, no other statistically significant differences in the magnitude of CT change were observed between glaucomatous and non-glaucomatous eyes at various time intervals, regardless of the presence of CME.

## Discussion

In this present study, SS-OCT was employed to evaluate changes in CT following phacoemulsification surgery in both glaucomatous and healthy eyes. To our knowledge, this is the first study to compare early macular CT changes (within the first three postoperative months) in glaucomatous eyes undergoing phacoemulsification using SS-OCT. Our findings indicate a consistent increase in macular CT measurements at one month after cataract surgery, observed in both glaucomatous and healthy eyes. Furthermore, this study aimed to assess the relationship between CT changes and the presence of postoperative CME within each group. In this context, glaucoma eyes exhibited a 31% increase in the odds of developing CME; however, the presence of postoperative CME did not increase the risk of subsequent changes in macular CT for either glaucomatous or healthy eyes. Notably, when CME was present, glaucomatous eyes exhibited a significantly larger magnitude of SFCT change in the first month (relative to baseline) compared to non-glaucomatous eyes.

According to existing literature, macular CT does not appear to be directly associated with glaucoma. Several investigations have indicated an association between glaucoma and a thinner choroid [29,30]. However, a counter perspective emerges from opposing studies, demonstrating that healthy and glaucomatous eyes have similar CT [31,32]. Mwanza et al. [31] found no correlation between the presence or severity of glaucoma and the thickness of the macular choroid, as measured by EDI-OCT. Additionally, the same researchers provided further evidence that the thickness of the choroid, measured using SD-OCT, was not reduced in the eyes with advanced open-angle glaucoma compared to either fellow normal eyes or the eyes with mild glaucoma [33]. Furthermore, Zhang et al. [34] reported that there was no significant association between glaucoma and CT as measured by SS-OCT. Nevertheless, it is important to note that older age and a longer axial length were correlated with a thinner choroid measurement, emphasizing the need to take these factors into account when interpreting CT measurements. Within our cohort, the preoperative CT, as measured by SS-OCT, was significantly thicker in glaucomatous eyes when compared to non-glaucomatous eyes across all five macular sectors (Table 1). This could be attributed to possible differences between glaucomatous and healthy subjects in physiological factors such as axial length, refractive status, body height, weight, body mass index, diastolic and mean blood pressure, and circadian change [35,36].

The impact of cataract surgery on CT remains uncertain due to conflicting evidence. The literature on this subject, using EDI-OCT, is limited and does not provide conclusive results. Some studies have reported an increase in CT following phacoemulsification cataract surgery [37], while others have found no significant change [6]. By using SS-OCT, Shahzad et al. [38] reported a gradual increase in SFCT that became significant one month after cataract removal in healthy eyes. The study conducted by Gudauskiene et al. [39] on healthy eyes concluded that, one month after cataract surgery, there was an insignificant subclinical increase in SFCT. However, a significant increase in SFCT was observed three months postoperatively. Our findings are consistent with previous research and contribute to the limited body of literature utilizing SS-OCT by demonstrating a significant increase in CT one month postoperatively. At one month, the SFCT and superior and inferior CT were significantly greater than the preoperative values in both the glaucoma and non-glaucoma groups. At three months, superior CT remained significantly increased in glaucomatous eyes, while superior, inferior, nasal, and temporal CT persisted significantly in non-glaucomatous eyes.

The pathophysiology of choroidal changes in the macular area, following cataract surgery, involves a complex interplay of inflammatory responses, the breakdown of blood-retina barriers, and morphologic changes in the choroidal layers; however, the exact mechanisms are not entirely understood. One hypothesis is that a post-inflammatory rupture in the outer blood-retina membrane, in association with the rupture of the inner blood-retina barrier, would enhance the intraretinal accumulation of fluid [40]. The surgical procedure of cataract surgery releases inflammatory mediators, such as prostaglandins, leukotrienes, and cytokines, which leads to the breakdown of the blood-aqueous barrier and the blood-retina barrier. This acute expression of pro-inflammatory genes and proteins has been found in the neurosensory retina and in the choroid of mice undergoing lens extraction, suggesting that the choroid may be involved in the development of postoperative changes [41].

Our results suggest that the presence of CME correlated with the earlier onset of significant changes in macular CT for both glaucomatous and non-glaucomatous eyes, evident at one week (Table 3). Moreover, when CME was present, the magnitude of SFCT change at the first month (relative to baseline) was significantly larger in glaucomatous eyes compared to non-glaucomatous eyes (Table 4). Pierru et al. [42] provided evidence that the increase in SFCT preceded the occurrence of pseudophakic CME, raising questions about the pathophysiological role of the choroid in the development of CME. Similarly, Fleissig et al. [43] concluded that choroidal thickness increased in the eyes with pseudophakic CME and decreased following edema resolution, which may strengthen the hypothesis of an inflammatory pathogenesis in pseudophakic CME. Additionally, Gudauskiene et al. [39] found that both the SFCT and the foveal retinal thickness (FRT) increased after phacoemulsification in non-glaucomatous eyes, as measured by SS-OCT; however, the study did not establish a direct correlation between the increases in SFCT and FRT. Interestingly, a study by Pakuliene et al. [44] investigated the late or chronic postoperative changes in the macula of non-glaucoma subjects and controlled and uncontrolled open-angle glaucoma patients using SS-OCT, six months after cataract surgery. The results indicated an increase in macular thickness across all groups. It was observed that the postoperative changes in choroidal thickness varied between the groups, possibly due to segmental choroid autoregulation. However, no association was found between glaucoma status and changes in choroidal or retinal thickness. Although the study did observe changes in choroidal thickness in different groups, these changes could not be associated with CME, as there were no cases of CME observed among the study participants.

Our findings indicate that in glaucomatous eyes, where CME was observed after cataract surgery, there may be a particular susceptibility of the choroidal vasculature to localized inflammation. Pilotto et al. [5] suggested that in non-glaucoma subjects, there could be a selective susceptibility to a localized inflammatory reaction induced by cataract surgery, observed in both the retina and the choroid. The early OCT angiography (OCTA) changes underlined the selective susceptibility of the deeper retinal plexuses (intermediate and deep capillary plexuses) to a localized inflammatory reaction induced by cataract surgery. Moreover, the appearance of hyperreflective retinal spots (HRS) at first in the inner retina and later in the outer retina seemed to confirm their inflammatory nature. Additionally, the study detected a second phase of choroidal thickening one month after surgery, which was coincident with the maximal increase of both retinal volumes and HRS. The authors hypothesized that activated retinal glial cells releasing cytotoxic substances, responsible for the inner blood-retina barrier breakdown, might be an additional factor contributing to the choroidal thickening detected at the first month. This hypothesis could also be applicable to our cohort of glaucoma patients, explaining the early choroidal changes observed in our study.

Our study has certain limitations. Firstly, it involved a relatively small sample size of glaucoma patients undergoing phacoemulsification, which could increase the risk of type 2 errors. The participants were selected using a convenience sampling method, with variations in sample size primarily dictated by the availability of participants at the time of the study. Nevertheless, the individuals enrolled in the study had homogeneous ethnicity and similar gender distribution, which reduced some confounding effects. Secondly, our study did not evaluate the severity of glaucoma or the specific treatments used. Glaucoma patients were undergoing treatment with topical IOP-lowering medications, which could potentially have influenced the CT measurements. It is known from previous research that CT measurements, obtained using SS-OCT, can show an increase as a response to reduced IOP [45].

## Conclusions

In conclusion, our study demonstrated a substantial increase in CT one month after phacoemulsification in both eyes with and without glaucoma. Furthermore, in cases where CME was present, glaucomatous eyes displayed a significantly more pronounced change in SFCT during the first postoperative month, as compared to the eyes without glaucoma. This observation implies a potential tendency for glaucomatous eyes to be selectively susceptible, in the early postoperative period, highlighting the need for additional research in this area. The findings of the present research are important for ophthalmologists as they provide a deeper understanding of the choroidal response to phacoemulsification. Such insights could potentially influence surgical decisions and strategies for glaucoma patient management.

## References

[1] Ho JW, Afshari NA (2015). The quest to optimizing cataract surgery outcomes. Curr Opin Ophthalmol.

[2] Benitah NR, Arroyo JG (2010). Pseudophakic cystoid macular edema. Int Ophthalmol Clin.

[3] Taravati P, Lam DL, Leveque T, Van Gelder RN (2012). Postcataract surgical inflammation. Curr Opin Ophthalmol.

[4] Kusbeci T, Eryigit L, Yavas G, Inan UU (2012). Evaluation of cystoid macular edema using optical coherence tomography and fundus fluorescein angiography after uncomplicated phacoemulsification surgery. Curr Eye Res.

[5] Pilotto E, Leonardi F, Stefanon G (2019). Early retinal and choroidal OCT and OCT angiography signs of inflammation after uncomplicated cataract surgery. Br J Ophthalmol.

[6] Falcão MS, Gonçalves NM, Freitas-Costa P (2014). Choroidal and macular thickness changes induced by cataract surgery. Clin Ophthalmol.

[7] Ohsugi H, Ikuno Y, Ohara Z (2014). Changes in choroidal thickness after cataract surgery. J Cataract Refract Surg.

[8] Agrawal R, Gupta P, Tan KA, Cheung CM, Wong TY, Cheng CY (2016). Choroidal vascularity index as a measure of vascular status of the choroid: measurements in healthy eyes from a population-based study. Sci Rep.

[9] Goldberg I (2003). Relationship between intraocular pressure and preservation of visual field in glaucoma. Surv Ophthalmol.

[10] Banitt M (2013). The choroid in glaucoma. Curr Opin Ophthalmol.

[11] De Moraes CG, Reis AS, Cavalcante AF, Sano ME, Susanna R Jr (2009). Choroidal expansion during the water drinking test. Graefes Arch Clin Exp Ophthalmol.

[12] Arora KS, Jefferys JL, Maul EA, Quigley HA (2012). The choroid is thicker in angle closure than in open angle and control eyes. Invest Ophthalmol Vis Sci.

[13] Hirooka K, Tenkumo K, Fujiwara A, Baba T, Sato S, Shiraga F (2012). Evaluation of peripapillary choroidal thickness in patients with normal-tension glaucoma. BMC Ophthalmol.

[14] Kubota T, Jonas JB, Naumann GO (1993). Decreased choroidal thickness in eyes with secondary angle closure glaucoma. An aetiological factor for deep retinal changes in glaucoma?. Br J Ophthalmol.

[15] Marangoni D, Falsini B, Colotto A (2012). Subfoveal choroidal blood flow and central retinal function in early glaucoma. Acta Ophthalmol.

[16] Yin ZQ, Vaegan Vaegan, Millar TJ, Beaumont P, Sarks S (1997). Widespread choroidal insufficiency in primary open-angle glaucoma. J Glaucoma.

[17] Spaide RF, Koizumi H, Pozzoni MC (2008). Enhanced depth imaging spectral-domain optical coherence tomography. Am J Ophthalmol.

[18] Hamzah F, Shinojima A, Mori R, Yuzawa M (2014). Choroidal thickness measurement by enhanced depth imaging and swept-source optical coherence tomography in central serous chorioretinopathy. BMC Ophthalmol.

[19] Adhi M, Brewer E, Waheed NK, Duker JS (2013). Analysis of morphological features and vascular layers of choroid in diabetic retinopathy using spectral-domain optical coherence tomography. JAMA Ophthalmol.

[20] Michalewska Z, Michalewski J, Adelman RA, Zawiślak E, Nawrocki J (2015). Choroidal thickness measured with swept source optical coherence tomography before and after vitrectomy with internal limiting membrane peeling for idiopathic epiretinal membranes. Retina.

[21] Saeedi O, Pillar A, Jefferys J, Arora K, Friedman D, Quigley H (2014). Change in choroidal thickness and axial length with change in intraocular pressure after trabeculectomy. Br J Ophthalmol.

[22] Sotiropulos K, Kourkoutas D, Chatzistefanou KI, Droutsas K, Moschos MM (2023). Changes in subfoveal choroidal thickness following uncomplicated cataract surgery: a scoping review. Cureus.

[23] Foster PJ, Buhrmann R, Quigley HA, Johnson GJ (2002). The definition and classification of glaucoma in prevalence surveys. Br J Ophthalmol.

[24] Kourkoutas D, Triantafyllopoulos G, Georgiou I, Karamaounas A, Karamaounas N, Sotiropulos K, Kapralos D (2022). Comparison of diagnostic ability between wide-field swept-source optical coherence tomography imaging maps and Heidelberg retina tomograph 3 Optic nerve head assessment to discriminate glaucomatous and non-glaucomatous eyes. Cureus.

[25] Hodapp E, Parrish RK, Anderson DR (1993). Clinical decisions in glaucoma. https://www.worldcat.org/title/clinical-decisions-in-glaucoma/oclc/27381902.

[26] Mrejen S, Spaide RF (2013). Optical coherence tomography: imaging of the choroid and beyond. Surv Ophthalmol.

[27] Huynh E, Chandrasekera E, Bukowska D, McLenachan S, Mackey DA, Chen FK (2017). Past, present, and future concepts of the choroidal scleral interface morphology on optical coherence tomography. Asia Pac J Ophthalmol (Phila).

[28] Kim SJ, Belair ML, Bressler NM, Dunn JP, Thorne JE, Kedhar SR, Jabs DA (2008). A method of reporting macular edema after cataract surgery using optical coherence tomography. Retina.

[29] Park HY, Lee NY, Shin HY, Park CK (2014). Analysis of macular and peripapillary choroidal thickness in glaucoma patients by enhanced depth imaging optical coherence tomography. J Glaucoma.

[30] Usui S, Ikuno Y, Miki A, Matsushita K, Yasuno Y, Nishida K (2012). Evaluation of the choroidal thickness using high-penetration optical coherence tomography with long wavelength in highly myopic normal-tension glaucoma. Am J Ophthalmol.

[31] Mwanza JC, Hochberg JT, Banitt MR, Feuer WJ, Budenz DL (2011). Lack of association between glaucoma and macular choroidal thickness measured with enhanced depth-imaging optical coherence tomography. Invest Ophthalmol Vis Sci.

[32] Maul EA, Friedman DS, Chang DS, Boland MV, Ramulu PY, Jampel HD, Quigley HA (2011). Choroidal thickness measured by spectral domain optical coherence tomography: factors affecting thickness in glaucoma patients. Ophthalmology.

[33] Mwanza JC, Sayyad FE, Budenz DL (2012). Choroidal thickness in unilateral advanced glaucoma. Invest Ophthalmol Vis Sci.

[34] Zhang C, Tatham AJ, Medeiros FA, Zangwill LM, Yang Z, Weinreb RN (2014). Assessment of choroidal thickness in healthy and glaucomatous eyes using swept source optical coherence tomography. PLoS One.

[35] Wei WB, Xu L, Jonas JB (2013). Subfoveal choroidal thickness: the Beijing Eye Study. Ophthalmology.

[36] Shao L, Xu L, Wei WB (2014). Visual acuity and subfoveal choroidal thickness: the Beijing Eye Study. Am J Ophthalmol.

[37] Noda Y, Ogawa A, Toyama T, Ueta T (2014). Long-term increase in subfoveal choroidal thickness after surgery for senile cataracts. Am J Ophthalmol.

[38] Shahzad R, Siddiqui MA, Zafar S, Kausar F, Shahzad MH (2018). Choroidal thickness changes following cataract surgery using swept source optical coherence tomography. Can J Ophthalmol.

[39] Gudauskiene G, Matuleviciute I, Mockute R, Maciulaityte E, Zaliuniene D (2019). Changes in subfoveal choroidal thickness after uncomplicated cataract surgery. Biomed Pap Med Fac Univ Palacky Olomouc Czech Repub.

[40] Bhagat N, Grigorian RA, Tutela A, Zarbin MA (2009). Diabetic macular edema: pathogenesis and treatment. Surv Ophthalmol.

[41] Xu H, Chen M, Forrester JV, Lois N (2011). Cataract surgery induces retinal pro-inflammatory gene expression and protein secretion. Invest Ophthalmol Vis Sci.

[42] Pierru A, Carles M, Gastaud P, Baillif S (2014). Measurement of subfoveal choroidal thickness after cataract surgery in enhanced depth imaging optical coherence tomography. Invest Ophthalmol Vis Sci.

[43] Fleissig E, Cohen S, Iglicki M, Goldstein M, Zur D (2018). Changes in choroidal thickness in clinically significant pseudophakic cystoid macular edema. Retina.

[44] Pakuliene G, Rylskyte N, Kuzmiene L, Siesky B, Verticchio A, Harris A, Januleviciene I (2023). Changes in macular thickness after cataract surgery in patients with open angle glaucoma. Diagnostics (Basel).

[45] Usui S, Ikuno Y, Uematsu S, Morimoto Y, Yasuno Y, Otori Y (2013). Changes in axial length and choroidal thickness after intraocular pressure reduction resulting from trabeculectomy. Clin Ophthalmol.

